# Advances in clinical examination of lacrimal gland

**DOI:** 10.3389/fmed.2023.1257209

**Published:** 2023-08-31

**Authors:** Yuan Lin, Yujie Zhang, Ke Shi, Huping Wu, Shangkun Ou

**Affiliations:** ^1^Xiamen Eye Center of Xiamen University, Eye Institute of Xiamen University, School of Medicine, Xiamen, Fujian, China; ^2^Fujian Key Laboratory of Ocular Surface and Corneal Diseases, Xiamen, Fujian, China; ^3^Xiamen Municipal Key Laboratory of Ocular Surface and Corneal Diseases, Xiamen, Fujian, China; ^4^Xiamen Municipal Key Laboratory of Ocular Diseases, Xiamen, Fujian, China; ^5^Department of Ophthalmology, The Affiliated Hospital of Guizhou Medical University, Guiyang, China

**Keywords:** lacrimal gland, clinical examination, advance, function, morphology, review

## Abstract

In humans, the lacrimal gland is located in the socket of the frontal bone above the outer orbital area. As an essential part of the eye surface, the gland is fixed to the orbital periosteum by connective tissue. The lacrimal gland passes through the outer tendon membrane, which divides the gland into larger orbital and minor eyelid glands. The lacrimal glands are the main contributors to tear film. They secrete electrolytes, proteins, and water to help nourish and protect the eye’s surface. Furthermore, clinically, lacrimal glands are associated with a variety of inflammatory reactions and immune factors and are also vulnerable sites for tumors. Changes in tear gland morphology or secretory function affect tear film stability and tear secretion quality. Various technological devices have been developed and applied to lacrimal glands. This article systematically reviewed the clinical examination of the lacrimal gland to help inform personalized strategies for the diagnosis of lacrimal gland-related diseases.

## Introduction

**Figure 1:**
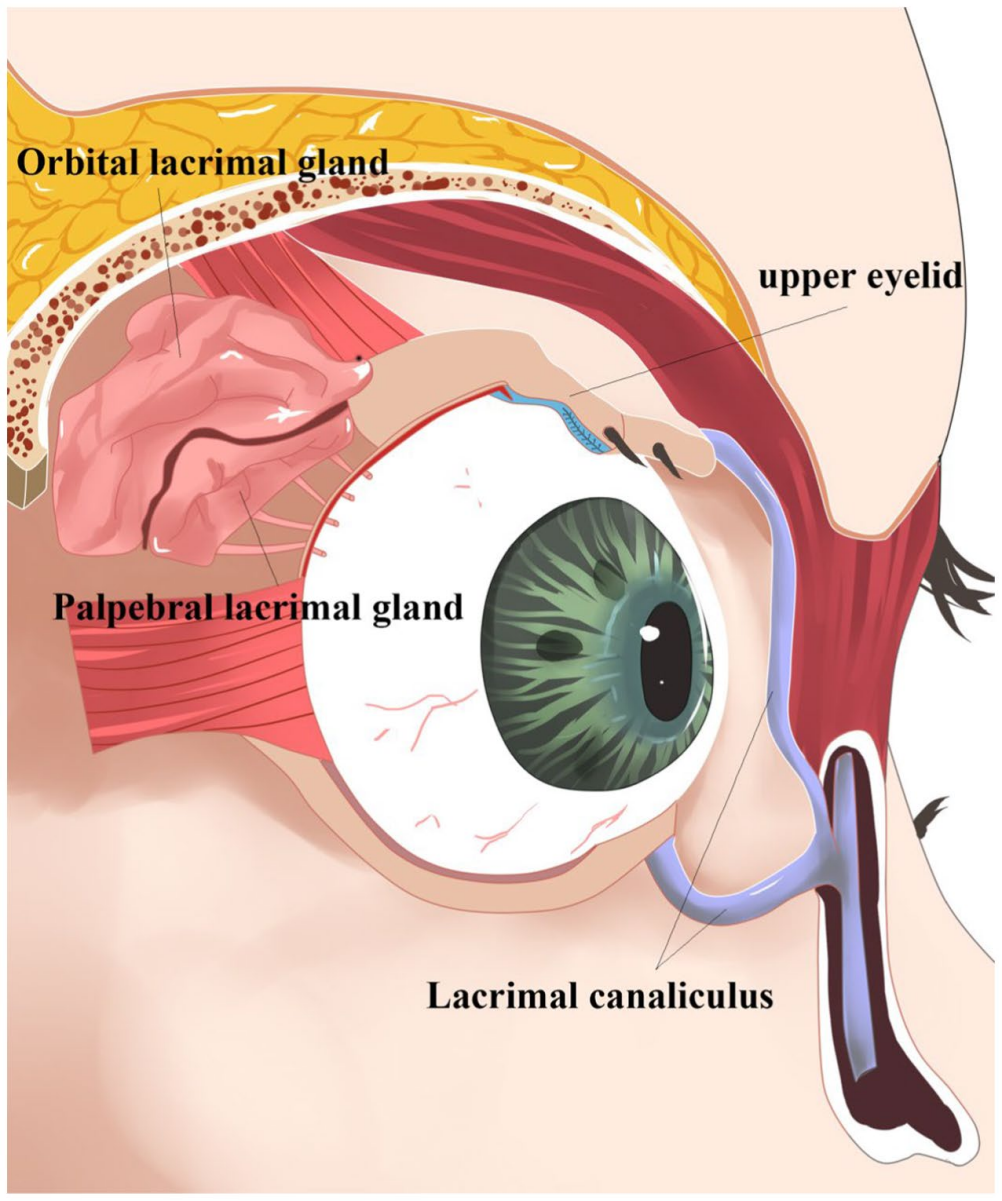
Oblique view of the right orbit. Oblique view of the right orbit showing the main lacrimal gland divided into the orbital lobe and palpebral lobe by the lateral horn of the levator aponeurosis. Note the excretory ducts coursing through the palpebral lobe and draining into the superior conjunctival fornix.

**Figure 2:**
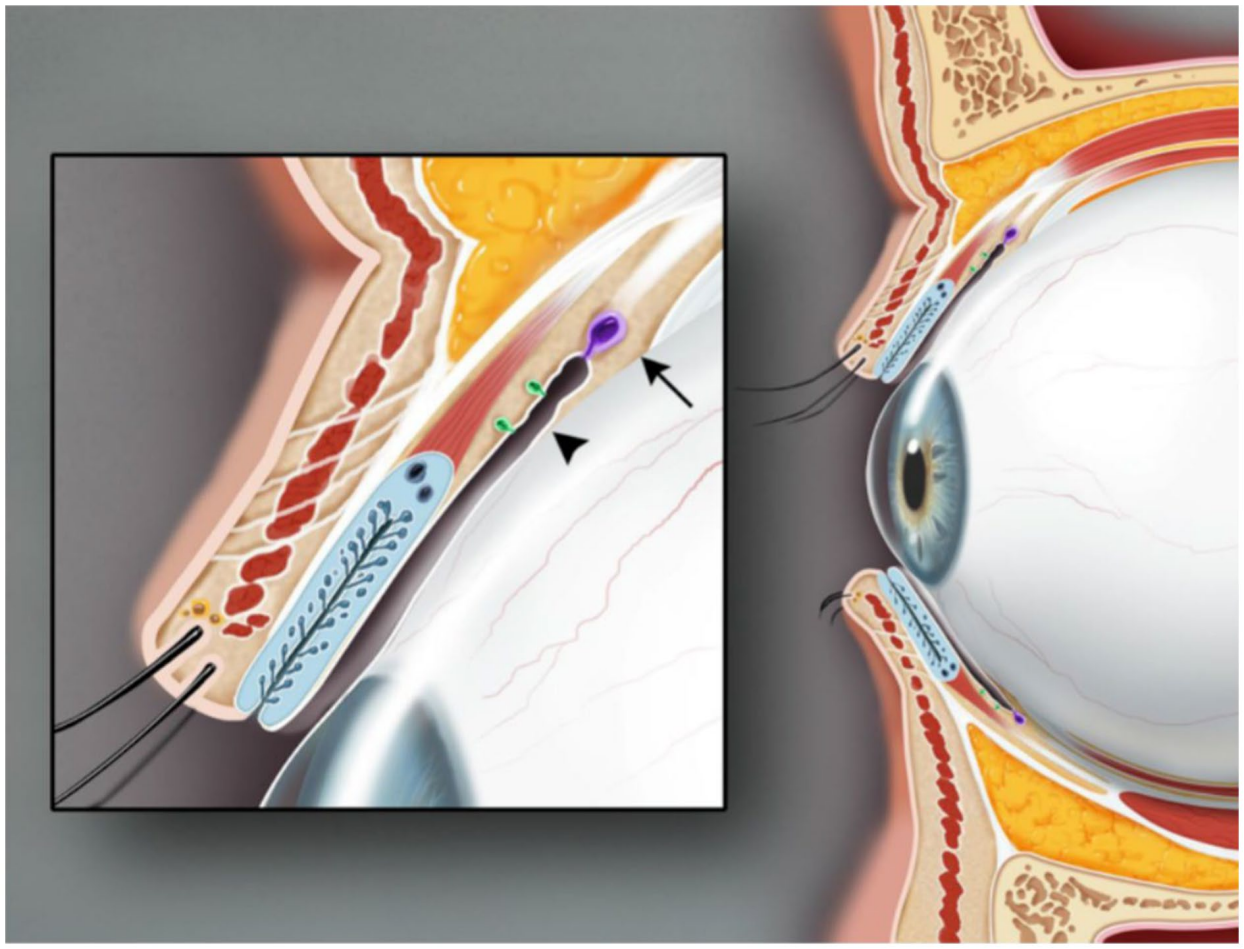
Sagittal view of the upper and lower eyelids. The glands of Krause (arrow) are located in the superior conjunctival fornix. The glands of Wolfring (arrowhead) are found at the nonmarginal border of the tarsal plate. Illustration from Conrady et al. (1).

The lacrimal gland, located above the orbit between the frontal bone and the eyeball (1) (Figure 1), is an essential part of the ocular surface and commonly participates in the protection and maintenance of the ocular surface. Like almonds, the lacrimal gland is divided into two parts. The orbital lacrimal gland, positioned in the upper outer quadrant of the eye’s orbit, is the larger and more significant of the two lacrimal glands in the eye. Its primary function is to produce the aqueous layer of tears. In contrast, the palpebral lacrimal gland, also referred to as the accessory lacrimal gland or Gland of Krause, is a smaller lacrimal gland located in the tarsal plates of both the upper and lower eyelids. When both the main and palpebral lacrimal glands collaborate, they produce an adequate amount of tears, keeping the eyes lubricated and protected, preventing dryness, and maintaining optimal ocular health. Tears secreted by ocular surface epithelial cells form a tear film that cleans the corneal surface to prevent harmful substance invasion and smooth the eyeball surface (1) (Figure 2). This tear film ensures a stable and optimal ocular surface microenvironment (2).

The lacrimal gland structure and function may contribute to the development of lacrimal gland diseases. Lacrimal gland disease is divided into two main categories: tumor and non-tumor diseases. Lacrimal gland tumors often include lacrimal gland pleomorphic adenomas and lacrimal adenoid cystic cancers. The typical appearance on image analysis of pleomorphic adenomas is a round to solid oval tumor with regular margins that occasionally causes bone remodeling and may have areas with calcification. In contrast, adenoid cystic carcinoma typically has irregular margins, appears nodular, infiltrates adjacent structures, and causes bone destruction (3). Non-tumor diseases include inflammatory lesions of the lacrimal glands and other lacrimal gland-related diseases, such as Sjogren’s syndrome, benign epithelial lesions of the lacrimal gland, and lacrimal gland lymphoma. The acute manifestations of lacrimal gland disease may be infectious or inflammatory. This condition is characterized by acute or subacute periocular pain, tenderness, upper eyelid oedema, and erythema. However, the subacute manifestations are more likely to be subversive. In addition, more rapid manifestations may include local lymphadenopathy, whereas purulent secretion or abscess formation suggests infection (4). Clinical evaluation of the tear gland and its function can aid in the diagnosis and treatment of lacrimal gland disease (see Table 1).

**Table 1:** The comparison of the lacrimal gland examination.

	Purpose	Advantage	Disadvantage
Anatomical tests: Palpation CT/ MRI Ultrasound	Check the morphology and tissue changes of the lacrimal gland	Intuitive and repetitive, it can compare the morphological and tissue changes caused by lacrimal gland diseases.	More detailed differential diagnosis of diseases with similar symptoms, medical history, and morphology is required, and pathological tissue examination requires multidisciplinary collaboration.
Invasive examination puncture	Check the pathology of lacrimal gland disease	If standard tests are inconclusive, a more precise and sensitive test may be needed to diagnose the issue.	To avoid causing harm and complications, it’s crucial to perform the examination using sterile techniques.
Tear function test: Schrimers Testing TMH	Check the amount of lacrimal gland secretion to check the lacrimal gland secretion function.	Fast, simple, and enables preliminary screening for some eye diseases with too much or few tears.	Tears secretion is less repetitive and affected by external stimulation.
Tear film stability tests: TBUT Tear osmolarity The component of the tear	The quality of the lacrimal gland secretion was determined by detecting the stability of the tear film.	The quality of tears that lacrimal glands secretion is known under the influence of eye surface diseases such as dry eye disease, Sjogren’s syndrome.	The environmental factors and the invasive inspections will affect the results.

TMH, tear meniscus height; TBUT, Tear break up time.

Recent studies have revealed a mutual relationship between the ocular surface epithelium and tear film (5). There are several eye diseases that can be linked to issues with the lacrimal gland’s secretory or excretory system. These issues may include inflammation, degeneration, and tumors (6). Up to now, it should combine the different examnation lacrimal gland to diagnose lacrimal gland diseases. Therefore, this review summarized and synthesized the clinical evaluation of lacrimal gland structure and function to aid in the diagnosis and treatment of lacrimal diseases.

## Anatomical tests

### Palpation

Palpation emerges as an indispensable technique in the examination of the lacrimal gland. By tactfully assessing the gland, eye care professionals can discern any modifications in its location caused by lacrimal adenitis or tumors. Lacrimal adenitis, characterized by the inflammation of the lacrimal gland, manifests as swelling, redness, and tenderness (7). The method of palpation aids in detecting the gland’s increased size and sensitivity, effectively differentiating it from other causes of eye discomfort.

Similarly, lacrimal gland tumors have the potential to disrupt the gland’s normal position, owing to their mass effect. Careful palpation allows for the identification of irregularities or firm masses within the gland, providing valuable information for the presence of tumors (3).

Lacrimal gland diseases or tumors can instigate abnormal eye positioning due to the displacement of the gland. This abnormality can result in strabismus, causing misalignment of the eyes. Consequently, an astute examination with palpation is essential for evaluating the eye’s position relative to the unaffected one and assessing any associated eye movement anomalies. Moreover, lacrimal gland diseases may present with lymphoproliferation or structural changes. The skillful application of palpation can aid in the detection of enlargement or nodules, which can be indicative of lymphoproliferative disorders or other structural abnormalities within the gland. While lacrimal gland diseases typically affect one eye, bilateral involvement is less common and often associated with orbital or periorbital changes. This includes conditions such as lacrimal muscle and lacrimal gland prolapse, blepharoptosis syndrome, or palpebral laxity (8). Conducting a comprehensive examination, including meticulous palpation, is crucial to accurately diagnose and manage these bilateral cases.

### Computed tomography/magnetic resonance imaging

A broad spectrum of lesions can affect the lacrimal gland and fossa. CT and MRI imaging technologies can provide crucial insights into the morphology of the ocular lacrimal gland. These advanced imaging techniques allow for a quick and comprehensive evaluation of the structure and shape of the gland. They are easy, convenient, and reliable methods for categorizing diseases affecting the lacrimal gland. In addition, they have added value for diagnosing lacrimal gland changes caused by systemic diseases and tumor-related lacrimal gland disease. The morphology of lacrimal glands can be well described using CT and MRI. Normal lacrimal glands are equally dense as muscle. Te medial border is delineated by the orbital fat, and the lateral border by the orbital bone. Calcification and bone changes can be observed by CT, and normal glands are symmetrically enhanced. The superior resolution of MRI allows for a better assessment of the degree of gland and periscapular involvement. Normal lacrimal glands show intermediate signals on both T1-weighted and T2-weighted imaging. This can be symmetrically enhanced after gadolinium administration (9).

In non-tumor diseases, CT and MRI technology have been applied to assess lacrimal gland function for the evaluation of conditions including Thyroid eye disease (TED) and Sjögren’s syndrome (SS). TED can decrease lacrimal gland secretion. CT and MRI can localize and characterize lacrimal lesions. Zhao et al. applied single-photon emission computed tomography (SPECT)/CT examination to demonstrate higher lacrimal gland volume involvement in patients with TED compared to that in healthy individuals (10). Gagliardo et al. showed that the measurement of lacrimal gland herniation by MRI can serve as a good and straightforward parameter to distinguish the activity of thyroid eye disease (11). CT and MRI have advantages in tumor-related lacrimal gland disease, especially MRI. MRI provides superior soft-tissue contrast resolution while avoiding the shortcomings of ionizing radiation. In addition, the pattern of contrast enhancement assists in confident preoperative diagnoses (12). Sufficient information can be obtained from the soft tissue in orbit by scanning lacrimal gland tumors in the occupying orbital lesions. This test can be used to obtain more detailed information to distinguish between benign and malignant tumors (13). Young et al. described the differences between malignant and benign epithelial lacrimal gland tumors, including irregular imaging, heterogeneity, calcification, and heterogeneity of bone invasion, providing excellent value for the accuracy of clinical diagnosis (14). However, similar overlapping characteristics are possible, highlighting the importance of imaging for distinguishing tumor types. Typical and common manifestations include spontaneous pain, paraesthesia, progressive eye protrusion with apparent displacement, eye compression deformation, decreased vision loss, diplopia, ptosis, and abnormal eye movement (15). The boundaries of the imaging manifestations are clear or unclear. Compression, bone infiltration, destruction of the bone adjacent to the lacrimal gland fossa, and accompanying calcification in some tumor lesions can cause difficulties in discrimination (16).

Imaging technology is a valuable tool for diagnosing lacrimal gland lesions; When assessing lacrimal gland lesions, it is crucial to consider multiple factors beyond clinical history and examination. In certain cases, histopathology remains the most dependable means of diagnosis.

### Ultrasound test

Ultrasound is a valuable and non-invasive way to assess lacrimal gland pathology in patients with lacrimal gland disease. By using high-frequency probes and careful examination techniques, doctors can identify structural abnormalities with precision, leading to accurate diagnosis and effective treatment of ocular conditions. To successfully visualize the lacrimal gland through ultrasound, high-frequency probes (> 22 MHz) are necessary for precise imaging (17). The lateral third of the upper orbital bone is a reliable anatomical reference point for locating the gland. Typically, ultrasound images show a small, homogeneous, hypoechoic region between the orbital bone and the sclera. Doctors commonly evaluate various parameters, including gland parenchymal visibility, size, uniformity, hypoechoic areas, hyperechoic points, fibrous gland appearance, and fat deposition. While the current dichotomous scoring system shows good to excellent intra-and inter-rater reliability for static images, it lacks sensitivity to subtle morphological changes (18). Future research should aim to develop more objective and comprehensive scoring systems for improved evaluation of lacrimal gland pathology in lacrimal gland disease patients using ultrasound.

## Invasive examination

### Puncture

There remain limitations in the diagnosis of diseases that affect the morphology of the lacrimal gland and imaging examination through clinical symptoms. Lacrimal gland-occupying lesions are challenging to distinguish from ocular surface diseases, such as inflammation. Inflammatory lesions are the most common cause of lacrimal gland enlargement, followed by lymphoproliferative disorders. Diagnosis usually requires a histopathological specimen, which is essential for identifying inflammatory lesions, benign and malignant lacrimal gland tumors, and metastatic disease (4). Some diagnoses of specific immune-related eye diseases rely on pathological biopsy. The diagnosis of lymphoproliferative diseases in the lacrimal area (19), inflammatory lessions (4), IgG4-related eye disease (20), and orbital lymphoma (9) must first exclude other diagnoses and are then determined based on clinical and pathological findings. Different lacrimal gland-occupying lesions have varying treatment options. Not suitable for occupying surgical lesions, lacrimal gland inflammation or lymphatic tumor through puncture biopsy can contribute to identification before treatment (21). Several different tear gland lesions can show clinical symptoms, with imaging results highly similar to those of lacrimal gland polyadenomas (4). Proper puncture biopsy may help reduce unnecessary surgical operations or complications caused by misdiagnosis (22). Imaging techniques have been used more frequently in tissue sampling; for example, MRI can locate the lesion range and initially detect and identify benign and malignant lesions. MRI can also be combined with fine-needle aspiration cytology (FNAC) to enhance diagnosis. Imaging techniques are easily and safely applied to lesions (23, 24). Ultrasound-guided puncture biopsy is safe and effective for adrenal lacrimal lesions, including identifying benign and malignant causes and reducing or avoiding invasive surgical biopsies in most patients (25).

Puncture biopsy provides clinical diagnosis and informs treatment. Patients also have a high tolerance to puncture biopsy, even with the cooperation of clinical ophthalmologists and pathologists. This examination necessitates meticulous adherence to aseptic protocols in order to mitigate any potential harm or complications resulting from medical intervention.

## Tests of lacrimal gland function:

### Schirmer’s test

The tear production rate is essential to eye health. The secretory function of lacrimal glands can be rapidly assessed using Schirmer’s test. Dry eye symptoms are likely to occur when tears are abnormally produced (26). Schirmer’s test is easy to apply. To evaluate the sufficiency of lacrimal production, a standardized paper strip is introduced into the inferior tear lake and draped over the lower eyelid. After a period of 5 min, the wetting length is measured and the final estimated wetted size provides a qualitative assessment of lacrimal production adequacy (27, 28). The consistency of Schirmer’s and red phenol tests is not satisfactory, and their sensitivity for detecting dryness is low (29). One study reported the correlation in results between the 1-min and 5-min tests. When there was no significant correlation, gaze was also affected during the test (30). Healthy subjects show better outcomes in Schirmer’s test compared to patients with SS-associated dry eye (31). In the absence of anesthesia, Schirmer’s test measures reflex tears, whereas the anesthesia test measures basal tear secretion (32). Schirmer’s test can assess the basis of lacrimal gland secretion but is affected by the environment, temperature, humidity, age, and other eye diseases (33).

### Tear meniscus height

TMH is the tear meniscus length band between the upper and lower eyelid margins. TMH can reflect normal and abnormal tear secretion and can help to diagnose dry eye disease (DED). It has shown a relatively high sensitivity and specificity. In addition, TMH is interrelated with Schirmer’s test (33). The measurement of TMH is noninvasive and repeatable. However, TMH is affected by forced blinks such as tear break-up time. Studies have shown that TMH measured after forced blinks increases significantly, while open eyes can also increase TMH (34). Moreover, the indoor temperature is negatively correlated with TMH: when the temperature increased, so did the TMH. A slit lamp is usually used to observe the level of lacrimal fluid at the junction of the lower eyelid margin light band and corneal conjunctiva surface light band. This level reflects tear secretion to some extent, similar to the tear secretion test. in DEWS II, a height ≤ 0.2 mm is indicative of dry eye (35).

### Optical coherence tomography

OCT is a noninvasive, high-resolution imaging technique based on low-coherence interferometry that can be used for clinical and experimental assessments of the ocular surface. OCT measures TMH with low variability and good reproducibility and repeatability (36). It can also provide *in vivo* cross-sectional images of the tissue structures. Evaluation of the tear film using OCT allows quantification of the tear meniscus dimensions and has the potential to measure tear film thickness (37) and obtain *in vivo* imaging of the tear film and epithelia. More advanced OCT, including a wider optical bandwidth, is needed for more accurate detection. Moreover, better lateral resolution can be obtained by using an alternative focusing lens for the image depth or via micro-optical OCT (38). Wang et al. have shown that TMH-OCT and TMA-OCT have high sensitivity and specificity for the diagnosis of mild dry eye, respectively (39). In conclusion, Optical Coherence Tomography (OCT) holds considerable value as a pivotal tool for assessing tear film instability and the underlying lacrimal gland dysfunction. With its efficient and expeditious screening capabilities, OCT shows promising potential in early detection and effective management of lacrimal gland dysfunction, thereby facilitating timely intervention and optimizing treatment outcomes for patients afflicted with lacrimal gland dysfunction disease. The non-invasive and quantitative attributes of OCT render it an invaluable addition to the armamentarium of diagnostic modalities in ophthalmology, advancing our comprehension of tear film dynamics and enabling personalized care for individuals experiencing lacrimal gland dysfunction.

### Detection of tear film stability

Tear film stability, which is essential for clinical measurement, can be used to diagnose and assess treatments for dry eye (40). When compression of the bulbar conjunctiva hurts conjunctival goblet cells, it affects mucin secretion and reduces ocular surface hydrophilicity. This can increase the tear film disequilibrium.

### Tear break up time

The Tear Break-Up Time (TBUT) test is a clinical method used to assess the stability and quality of the tear film on the surface of the eye. The test measures the duration it takes for the tear film to break up or become unstable after a blink. The tear film consists of three layers, namely, the aqueous, mucin, and lipid layers. The aqueous layer, which is the largest component and produced mainly by the lacrimal gland, helps maintain the eye’s moisture. The mucin layer, secreted by goblet cells on the conjunctiva, ensures that tears spread evenly over the cornea, while the lipid layer, produced by the meibomian glands in the eyelids, reduces tear evaporation (41).

During the TBUT test, a fluorescein dye is applied to the eye’s surface and evenly distributed by blinking a few times. The patient is then asked to keep their eyes open without blinking while the examiner observes the tear film using a slit lamp microscope equipped with blue light. The test identifies dry spots or areas of the tear film breaking up, and the duration from the last blink to the appearance of these signs is recorded as the TBUT (42). A normal TBUT is usually around 10 to 15 s. A shorter TBUT indicates tear film instability, which may be due to conditions such as dry eye syndrome, meibomian gland dysfunction, or other ocular surface disorders. A longer TBUT is less common but may occur in some conditions (35).

A stable tear film is vital for keeping the ocular surface smooth and optically clear. A decrease in TBUT could suggest that a lack of tear film stability is linked to reduced tear volume. However, whether there is a barrier to lacrimal gland functional secretion still needs to be comprehensively evaluated in conjunction with other assessments.

### Tear osmolarity

Tear osmolarity is a single biophysical measurement that captures the balance between the input and output of tear film dynamics. The dynamic input and output of tears from tear osmolarity are essential factors in maintaining the dynamic balance of tears. The vigorous circulation of the incisions depends on the composition of each link. Changes in lacrimal gland function cause a dynamic tear imbalance. Therefore, data on tear osmolarity can help the clinical understanding of lacrimal gland function and stability of tear circulation (43).

Tear osmolality is an indicator of ocular surface injury and inflammation. Increased tear osmolarity is most likely to occur due to damage to the lacrimal gland (44). Tear osmolality and disease severity are strongly correlated. High permeability is associated not only with dry eye but also with disease severity and dehydration, diabetes, and thyroid eye disease (45). For example, dry eye involves progressively elevated tear osmolarity and worsening disease severity. Increased tear osmolarity, leading to ocular surface inflammation, is an essential factor in the pathogenesis of DED. Environmental factors should also be included in the reference. Smoking can also increase tear film osmolarity and impair the ocular surface and tear film function (46). Tear osmolarity is positively correlated with eye diseases and significantly decreased lacrimal gland secretion function. Tear osmolarity may also be an indicator for the classification of eye tears in patients with insufficient diseases. It should be used in other clinical trials, or in cases with a conflict between signs and symptoms (44, 47).

## Tear components test

### Lactoferrin

Lactoferrin is a multifunctional iron-binding glycoprotein expressed and secreted by glandular epithelial cells. LF can decay oxidative stress damage and inhibit inflammatory mediators in lacrimal glands (48, 49). LF affects physical metabolism and the lacrimal gland and is related to tear secretion. LF is vital for maintaining ocular surface health, regulating ocular inflammation, and regulating cell growth (50). LF is an abundant protein in tears, and the LF level is used to indicate the possible presence of dry eye diseases. However, the secretion of tears and the components of LF decrease with age. LF measurement is positively related to Schirmer’s test, representing the determination of dry eye disease level and providing diagnostic information (51–53).

### Tear ferning test

The tear ferning test is an easily performed test used to observe the tear fern pattern. A sample of tears dried on a glass microscope slide produces a crystallization pattern. Various ferning patterns can be observed, depending on the composition of the tear film. Healthy tear samples produce fully dense ferning patterns, while the ferning pattern is fragmented or absent in dry eye samples. Tear ferning is associated with the secreted aqueous volume, with some correlation with tear film stability (54). One study showed the resilience of the chemical composition of tears before and after stimulation due to the steady concentration of electrolytes and lipids within the tear film for different tear states. Thus, the tear ferning test may be clinically useful to assess the quality of lacrimal secretions (55).

The tear ferning test is used as an auxiliary diagnostic method for evaluating patients with dry eye disease. However, high humidity conditions change the normal tear ferning mode; thus, stable conditions are required to obtain repeatable results in the tear ferning test (56). The tear ferning pattern of dry eyes can improve after artificial tears are used (54). Diabetes causes decreased tear film stability, and the tear ferning test can estimate tear film quality in patients with diabetes and dry eye (57). These tests also provide information about the tear film environment in patients with dry eye. However, it is difficult to obtain pure results as electrolytes or macromolecules in tears can affect the results. In addition, the classification of ferning test results lacks standards (54). The tear ferning test has good repetition in healthy subjects (57), but varies significantly with changes in lacrimal gland function or abnormal tear quality. Therefore, this test must be combined with other tests such as Schirmer’s test.

### IgG4

Tears contain multiple anti-microbial proteins. The immunoglobulins (Igs) in normal human tears are mainly soluble IgA and IgG. Individuals can show significant differences in Ig content. Adults have normal serum IgG4 levels of <86.5 mg/mL. However, higher levels (≥135 mg/dL) may indicate lacrimal gland disease (58). IgG4-associated lacrimal adenitis shows typical lacrimal gland swelling, elevated serum IgG4 levels, and substantial IgG4-positive plasma cell infiltration (20).

## Discussion and insight

Tears are mostly produced by the lacrimal gland. Tears consist of mucus, slurry, and lipids and have multiple functions that form a tear film on the eye surface to prevent dust, smoke, and microorganisms from directly invading the eye. Tears also remove inflammatory products in the conjunctival capsule, microorganisms, and other foreign bodies, and also shed cells. Tears contain various antimicrobial substances such as antibodies, lysozyme, and lactoferrin, which can prevent the invasion of pathogenic microorganisms into the cornea and conjunctiva. In addition, tears play a role in healthy eye surface tissues and exclude metabolites.

Changes or damage to the lacrimal gland can lead to a decreased eye surface state. Uncomfortable or dry eyes can be attributed to the quality of lacrimal gland secretion. The tear film consists of an aqueous-mucin layer containing fluid and soluble factors produced by the lacrimal glands, as well as mucin secreted by the goblet cells covered by a lipid layer. Tears contain proteins, glycoproteins, and lipids to maintain a stable ocular surface. Tear tests are a valuable tool for evaluating the performance of the lacrimal gland and identifying various eye conditions. Additionally, they serve as a means of monitoring systemic illnesses and tracking the impact of diagnostic and therapeutic medications. As such, tear tests play a crucial role in promoting optimal eye health and overall wellness.

The lacrimal gland is located in the lacrimal fossa. It is adjacent to the tendons of the superior and lateral rectus muscles and separates them from the globe. The gland is deeply indented by the lateral border of the levator palpebrae superioris, dividing the gland into larger orbital and smaller palpebral lobes. The occurrence and development of lacrimal gland disease can change the typical structure of the eye table. Clinical observation of the lacrimal gland area can reveal lacrimal gland disease. Lacrimal gland disease of the orbital shows characteristic signs, such as lacrimal gland inflammation, upper eyelid swelling, typical upper eyelid drooping, slight eyeball downturn or inward, tears, or purulent secretions. However, evaluating lacrimal gland status through clinical symptoms can help with the differential diagnosis of lacrimal gland diseases. Lacrimal gland tumors (3) can also cause swelling of the tear gland and skin. Imaging examination can clearly show the tumor size and degree of invasion of the lacrimal gland socket, such as systemic immune-related lacrimal gland disease or systemic diseases that require identification through biopsy.

Tears, including basal and reflex secretions, are primarily secreted by the lacrimal gland. The secretory activity of the former has no innervation and is constant day and night. The latter secretion is innervated by sympathetic, parasympathetic, and sensory nerves (59). Tears produce a smooth corneal epithelial surface, and moist and nourishing conjunctival and corneal epithelial cells remove dust and debris and prevent pathogens, thus improving the optical characteristics of the eye surface (60). Consequently, the nerve reflex produces a tear secretion effect when the body is under local, systemic, or mental stimulation.

Schirmer’s test, which describes tear production, remains the most widely used clinical test. However, this test is an invasive examination and its measurement may contain degeneration. The Results are affected by sex, age, and environment (32). After anesthesia patients show better stability and eyes receiving unilateral anesthesia will also affect the results of bilateral trials. Thus, local anesthesia affects reflex secretion (61). Furthermore, measurements of TMH have achieved non-invasive and reproducible effects using high-resolution OCT imaging techniques that show relatively high sensitivity and specificity (62). Currently, the oculus kinematograph is applied as a new method for TMH measurements, with high precision, good repetition, and without trauma (63).

Changes in tear composition and stability can lead to eye stimulation, corneal epithelial diseases, neurological diseases, and blurred vision (64). Environmental temperature significantly affects tear film characteristics, with indoor and outdoor factors producing different results. A dry environment causes increased tear film evaporation and reduced tear flow rate and may lead to dry eye (65). Impairment of lacrimal gland function can lead to debilitating diseases such as water deficiency, untreated dry eye, corneal ulcers, and vision loss. Various research methods, including corneal topography, interferometry, tear film meniscus measurement, evaporation rate, and osmolarity, have been developed to evaluate the structural and functional characteristics of tear films (66).

**Figure 3:**
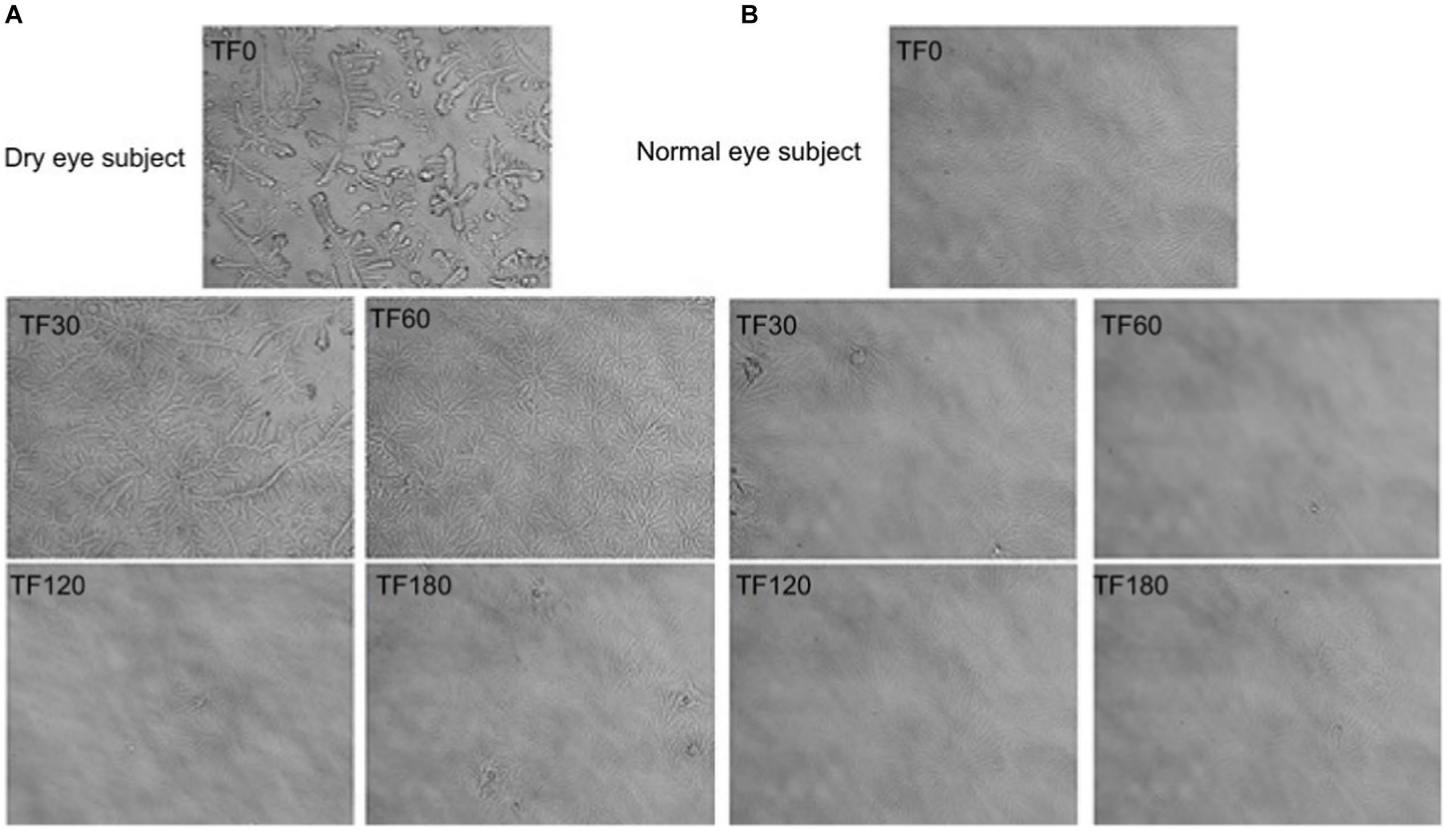
Tear ferning (TF) images obtained before and after application of eyedrops in the right eye of **(A)** a dry-eye subject and **(B)** a normal eye subject. TF0-180, TF scores obtained 0–180 min after application of eyedrops. Illustration from Alanazi et al. (67).

Laboratory tests of tear gland function are rare in clinical practice. Eye diseases with functional changes in the lacrimal glands are diagnosed based on symptoms, signs, medical history, and other examination techniques. The tear ferning test is a laboratory trial used to evaluate the severity of dry eye in patients (67) (Figure 3). While easy to perform, the test needs to be applied clinically with other examination methods (54). Despite a large amount of available data and many biomarkers targeting several ocular and systemic diseases proposed (68), the degree of translation into well-characterized and clinically valuable tools is largely insufficient. Single-Tear Proteomics provides new methods and precision medicine ideas in the new lacrimal gland proteomics (69). However, translating the results of molecular biology and omics studies of eyes and tears into clinical ophthalmology is a just starting complex process.

The eye surface microbiome and human eye surface tissue cells work in harmony to maintain eye surface homeostasis and promote optimal eye health. The normal eyes of healthy people have a stable microbial ecology, whereas people with eye diseases show a more diverse eye surface microecology (70). Examination of tear microflora revealed different surface distributions between ocular diseases and normal flora. Therefore, ocular disease may alter the secretion mass of the lacrimal gland and tear secretion formed by inflammatory exudates.

Elevelevated oxidative stress may lead to impaired lacrimal glands and induce the accumulation of carbon-sylated proteins in the lacrimal glands (71), thus leading to multifocal inflammation and fibrosis around the lacrimal acinar cells. Furthermore, studies indicated altered regulatory effects of pro-inflammatory and protective proteins in tears of Sjogren’s syndrome (72, 73) and thyroid-associated orbitopathy (74, 75), reflecting both autoimmune and inflammation-induced lacrimal gland dysfunction. Studies have pointed out that eye diseases can also participate in inflammation-related pathways to cause lacrimal gland function changes (76). Tear membrane damage and lacrimal drainage obstruction after trauma are also the causes of the changes in lacrimal gland function (77).

In molecular biology, screening new biomarkers requires early disease diagnosis and timely action to prevent more severe stages (78). In addition, finding ways to reduce the oxidative stress-related damage (79) of the lacrimal gland and retain the lacrimal gland function can significantly improve the tear volume and increase the amount of tear protein secretion. However, it has not been applied to clinical trials on a large scale. Still, we look forward to the future to develop various therapeutic interventions to treat lacrimal gland function impairment.

## Conclusion

The lacrimal gland is a critical component of the eye’s surface, and any changes in its long-term function or form can lead to damage. Though patients may not experience discomfort, it is essential to undergo practical and clinically active examinations related to the lacrimal gland to diagnose, treat, and prevent any diseases. While numerous methods are available to examine the function, morphology, and composition of the lacrimal fluid, there is a lack of methods that meet specific needs. Thus, multiple examination methods are necessary to ensure a more comprehensive evaluation of the lacrimal glands.

## Method of literature search

The literature review was conducted in a comprehensive PubMed search without date restrictions at the end of April 2022 for references in English related to the following keyword: “lacrimal gland” in combination with “dry eye” or “ophthalmology” or “lacrimal” or “tear.” Articles were excluded if they were not referenced in English. Emphasis was given to RCTs, meta-analysis, original research, and prospective studies.

## Author contributions

YL: Data curation, Investigation, Methodology, Writing – original draft, Writing – review & editing. YZ: Data curation, Formal analysis, Methodology, Writing – original draft. KS: Data curation, Formal analysis, Software, Writing – review & editing. HW: Project administration, Resources, Validation, Visualization, Writing – review & editing. SO: Conceptualization, Data curation, Formal analysis, Funding acquisition, Investigation, Methodology, Project administration, Resources, Software, Supervision, Validation, Visualization, Writing – original draft, Writing – review & editing.

## Funding

The author(s) declare financial support was received for the research, authorship, and/or publication of this article.

This study was supported by the National Natural Science Foundation of China (82101084, 82060173), China Postdoctoral Science Foundation (2021M69898), the Fujian Provincial Science Fund for Distinguished Young Scholars (2023J06053), and funding from the Xiamen Science and Technology Program for Public Wellbeing (3502Z20209183, 3502Z20224ZD1209, 3502Z20224ZD1210). The funders had no role in the study design, data collection and analysis, publishing decision, or preparation of the manuscript.

## Conflict of interest

The authors declare that the research was conducted in the absence of any commercial or financial relationships that could be construed as a potential conflict of interest.

## Publisher’s note

All claims expressed in this article are solely those of the authors and do not necessarily represent those of their affiliated organizations, or those of the publisher, the editors and the reviewers. Any product that may be evaluated in this article, or claim that may be made by its manufacturer, is not guaranteed or endorsed by the publisher.
